# Factors affecting COVID-19’s mental health impact on college students in the southeastern United States

**DOI:** 10.3389/fpubh.2023.1225686

**Published:** 2023-11-15

**Authors:** Tiffany Chenneville, Kemesha Gabbidon, Campion Zharima, Stefanie Hornschuh, Janan Janine Dietrich

**Affiliations:** ^1^Department of Psychology, University of South Florida, St. Petersburg, FL, United States; ^2^Perinatal HIV Research Unit, Faculty of Health Sciences, University of Witwatersrand, Johannesburg, South Africa; ^3^Health Systems Research Unit, South African Medical Research Council, Belville, South Africa; ^4^African Social Sciences Unit of Research and Evaluation (ASSURE), Faculty of Health Sciences, University of Witwatersrand, Johannesburg, South Africa

**Keywords:** COVID-19, mental health, college students, southeastern, United States

## Abstract

Emerging evidence confirms COVID-19’s negative impact on college students’ mental health; however, more research is needed to identify factors that promoted or hindered college students’ mental health early in the pandemic. This exploratory study addressed this need. Participants were 697 students attending a large, state, urban university in the southeastern United States. Using a cross-sectional survey design, participants completed an anonymous, online survey assessing socio-demographic variables, mental health issues, and activities during the lockdown period in 2020. Findings suggest college students in the southeastern US who were women or transgender men and had pre-existing mental health conditions, fewer routine activities, and high exposure to COVID-19 news reported more mental health problems early in the pandemic. Students who exercised daily had fewer symptoms of anxiety and post-traumatic stress than students who exercised monthly, but there were no differences in emotional responses to COVID-19 based on exercise frequency. Tailored strategies to address college students’ needs in response to the current or future pandemics are needed and should take into consideration factors that promote or hinder mental health. Patient or Public Contribution: College students were participants in this study. College students who were not participants in this study assisted with the implementation of this study.

## 1 Introduction

Like other pandemics, Coronavirus Disease 2019 (COVID-19) has threatened mental health around the world (1–5). The high prevalence of deep traumas in societies overwhelmed by natural disasters and global pandemic diseases, such as COVID-19, and the resulting increase in psychological stress and psychiatric disorders has been documented (6–8). An estimated 16% of the world’s population was affected by mental health disorders before the COVID-19 pandemic including high rates of depression, anxiety, and post-traumatic stress (9). Although it is still too early to accurately estimate the lasting mental health impact of COVID-19, its threat to global mental health is well established (10). Shigemura et al. (10) and Hossain et al. (11) described COVID-19’s potential effects on physical and mental health in terms of extreme fear of illness and anxiety driven by risk perceptions and poor health. However, the indirect effects of COVID-19 also have negatively affected mental health (e.g., job loss, financial issues, increased caregiving) (12–15). Further, a systematic review of studies addressing the mental health consequences of COVID-19 provides evidence of decreased psychological well-being among the general population in response to the pandemic (3). Of note, most studies included in the review were conducted in China.

Young people are likely to be susceptible to mental health problems associated with the COVID-19 pandemic including anxiety, depression, and stress symptoms (16). This may be especially true for college students (17), a population already at risk for mental health and substance use issues due to academic stress, family separation, and the need to juggle multiple responsibilities (e.g., work, school, family, and friends) (18).

Indeed, COVID-19’s mental health impact on college students has been studied in countries across the world. Findings from a global systematic review and meta-analysis of studies published through September 2020 documented COVID-19’s negative impact on college students’ mental health early in the pandemic (5). In another systematic review, Wang et al. (4) found symptoms of anxiety, stress, and depression were higher among non-Chinese students than Chinese students. In a cross-sectional survey study, Wang et al. (19) found most students attending a university in the southwestern United States reported pandemic-related increases in, and difficulty coping with, stress and anxiety. Son et al. (20) reported similar findings in the same region. In a comparison of pre-pandemic to pandemic time points, Frazier et al. (21) found college students at a midwestern university in the US had higher depression and stress rates during the pandemic than they did several years before the pandemic’s onset. Studies in Bangladesh (22) and Italy (23, 24) showed similar findings.

Fewer studies have examined specific factors associated with COVID-19’s mental health impact on college students, although the literature in this area is growing. Among a French sample of college students, Wathelet et al. (25) identified multiple factors affecting COVID-19’s mental health impact including gender, income, housing, psychiatric history, social isolation, low quality COVID-19 information, and COVID-19 symptoms. Using survey data from students attending large, public, research universities across eight US regions, Soria and Horgos (26) found some marginalized groups were at increased risk for depression and anxiety symptoms during COVID-19 as were students who were disabled or caretakers. Financial stress, food insecurity, housing issues, and academic stressors also affected mental health issues among students in Soria and Horgos’ study (26).

The impact of COVID-19 on college student mental health in the southeastern US is understudied despite this region being considered especially vulnerable to the pandemic’s negative impact. Americans living in the south are more likely to live in poverty and be uninsured, which contributes to poor health outcomes (27). Health disparities and systemic racism also contribute to COVID-19’s negative impact in this region given that many racial and ethnic minorities live in what is often called the Deep South. These factors, combined with the political climate in this region, negatively affect healthcare access, including mental health treatment (28). According to the Centers for Disease Control (CDC) (29), 13 of 16 southern states were ranked in the highest 50% of COVID-related deaths in the US. It is difficult to compare COVID-19 rates and outcomes in the southeastern US to the rest of the world, in part, because of problems with tracking in the southern states. For example, tracking of up-to-date vaccinations among state residents designated as southern states by the US census bureau (30) are among some of the lowest in the nation (31). However, World Health Organization (32) data suggests that, globally, the US is responsible for 16% of all COVID-related deaths and 13% of infections, though the vaccination rate of the US closely compares with global vaccine rates.

As demonstrated above, emerging evidence confirms COVID-19’s negative impact on college students’ mental health. Information about the circumstances associated with COVID-19’s mental health impact also is increasing. However, more research is needed to better understand what factors promote or hinder college students’ mental health so that prevention strategies and interventions can be tailored to meet students’ needs and inform planning for future pandemics. This kind of research is particularly important in the southeastern US given this region’s vulnerability to the negative outcomes related to the pandemic, as described above. To address this need, this exploratory study’s primary purpose was to assess factors associated with COVID-19’s mental health impact early in the pandemic on college students at a large university in the southeastern US. Specifically, this study aimed to examine the relationship between socio-demographic variables, activities during the COVID-19 lockdown, and mental health symptoms (e.g., depression, anxiety, and post-traumatic stress).

## 2 Materials and methods

### 2.1 Study design

We used a cross-sectional survey design.

### 2.2 Participants and setting

Participants were 697 students enrolled in a summer course in 2020 at one of three campuses that are part of a large, research intensive, public university in the southeastern US. Only students 18 and older could participate. Minors were excluded.

Only a few days before data collection, the university consolidated, prior to which the campus where data was collected was a separately accredited institution within the larger university system. This context is important for explaining procedures and understanding response rate (see below). The student population is approximately 50,000 across the three campuses, but approximately 4,000 on the campus where data was collected. The demographic makeup of students is predominantly white (63.6%), non-Hispanic (82.7), women (63.4%) aged 18–24 (64.8%) (33), which is representative of the larger population of college students in the US (34). The university offers student mental health services, including counseling, through wellness centers located on each campus. Services were offered virtually during the pandemic. Of note, in the state where data was collected, a state-wide stay-at-home mandate was issued on April 4, 2020, and lifted on April 30, 2020. City and county stay-at-home mandates within the state varied but had ended prior to data collection.

### 2.3 Procedures

This study was reviewed by the University of South Florida Institutional Review Board and determined exempt given its use of anonymous data. Following university approvals, the campus registrar sent an invitation email to 3,791 students on July 5, 2020, to complete an online survey administered through Qualtrics survey software. As a quality control measure, Qualtrics survey options were set to prevent multiple submissions by the same respondent, thus ensuring each participant completed the survey only once. The survey was closed on August 7, 2020. Due to consolidation issues beyond the investigators’ control, a reminder email was never sent, which may have negatively affected response rate. Although measures were not taken to mitigate this issue, the response rate was approximately 17%, which falls within a typical range for online survey response rates.

Participation was completely voluntary. Responses were anonymous. After an introduction to the study, participants were notified that by completing the survey they were agreeing to participate. Participants were told they could discontinue the survey at any time. At the survey’s end, participants received information about how to access mental health services, if needed, through the university wellness centers. Using a systematic or quasi-random selection procedure, every 10th participant who completed the survey received a $10 Amazon gift card up to 500 participants. To limit sampling bias, participants were blind to survey completion numbering. Contact information for incentives was gathered after the survey was completed and was not linked to survey data.

### 2.4 Measures

The measures used to assess socio-demographic variables, mental health, and activities during lockdown early in the pandemic are described below.

#### 2.4.1 Socio-demographics

A socio-demographic questionnaire gathered data on age, gender identity, race, ethnicity, degree program (undergraduate versus graduate level), monthly income, mental health history (including diagnoses), daily cell phone use (in hours), COVID-19 testing, food security, and living situation (including any housing disruptions) during the height of the pandemic. Race and ethnic categories were determined by the US Census Bureau’s characterization (35); however, authors recognize understanding of racial and ethnic identity varies among cultural groups, see Table 1.

**Table 1:** Sociodemographic characteristics of study participants, *N* = 697.

	*n*	%
Age		
18–19 years	169	24.2
20–29 years	429	61.5
30–39 years	63	9.0
40–49 years	19	2.7
50–59 years	11	1.6
60 or older	6	0.9
Gender^a^		
Men	147	14.7
Women	526	75.5
Transgender men	5	0.7
Transgender women	0	0
Prefer not to say	4	0.6
Prefer to self-identify	12	1.7
Missing	3	0.4
Race^b^		
Black/African American	70	10.0
White	554	79.5
Asian	50	7.2
American Indian/Alaskan Natives	7	1.0
Native Hawaiian and Pacific Islander	6	0.9
Other	57	8.1
Ethnicity		
Hispanic	122	17.5
Non-Hispanic	572	82.1
Missing	3	0.4
Degree program		
Undergraduate certificate	105	15.1
Graduate certificate	17	2.4
Bachelor’s degree	448	64.3
Master’s degree	75	10.8
Doctoral degree	20	2.9
Other	4	0.6
Non-degree seeking	11	1.6
Missing	17	2.4
Monthly income		
$0–$500	286	41.0
$501–$1,000	145	20.8
$1,001–$1,500	88	12.6
$1,501–$2000	46	6.6
$2001–$2,500	35	5
$2,501–$3,000	16	2.3
$3,000+	55	7.9
Missing	26	3.7
Previously diagnosed with emotional difficulty or mental illness		
Yes	268	38.5
No	388	55.7
Missing	41	5.9
Phone use in hours		
0-2 h	49	7.0
2-4 h	158	22.7
4-6 h	195	28.0
6–8	114	16.4
Always	103	14.8
Tested for COVID		
Yes	19	2.9
No	646	92.7
Missing	32	4.6
Did you have to move out of the residence halls?		
Yes	96	13.8
No	80	11.5
I do not live in the halls	493	70.7
Missing	28	4.0

^a^Among the 12 participants who opted to self-identify gender, five reported being non-binary while others reported concerns about categorizing gender.

^b^Several participants skipped the question on race and chose to identify as other in the “racial” category and Hispanic in the ethnic category rather than indicating whether they were White Hispanic, Black Hispanic, or other racial and ethnic combinations.

#### 2.4.2 Mental health

Mental health was assessed using: (1) the General Health Questionnaire-12; (2) the Primary Care Post Traumatic Stress Disorder Screen; and (3) four items assessing emotional responses to the COVID-19 pandemic.

#### 2.4.3 General Health Questionnaire-12

The 12-item General Health Questionnaire (GHQ-12) (36) was used to assess mental health during the pandemic. The GHQ-12 includes items such as *Have you recently been feeling unhappy and depressed?* Participants responded to items using a four-point Likert scale. Response options varied by question. For some items (e.g., *Have you recently been able to face up to your problems?*), response options were 0 = *more than usual,* 1 = *same as usual,* 2 = *less than usual,* and 3 = *much less than usual*. For other items (e.g., *Have you recently been losing confidence in yourself?*), response options were 0 = *not at all,* 1 = *no more than usual,* 2 = *more than usual,* and 3 = *much more than usual.* Scores ranged from 0 to 36, with higher scores indicating more severe psychological distress. Cronbach’s alpha in our sample was 0.899. Total scores under 16 indicate no stress, scores 16–20 indicate stress, and scores >20 indicate great psychological distress (36).

#### 2.4.4 Primary care post traumatic stress disorder screen

Symptoms of posttraumatic stress disorder (PTSD) were assessed using six questions adapted from the Primary Care PTSD Screen (PC-PTSD). The PC-PTSD is considered a valid, reliable instrument and is recommended by the American Psychiatric Association (37). A Cronbach’s alpha of 0.76 was reported for the original sample (37). The original PC-PTSD survey has general questions on frightening or traumatic events. Items were adapted to make the instrument more specific to the COVID-19 pandemic. For example, participants were asked *Do you have intrusive thoughts about the COVID-19 pandemic that cause repeated, distressing memories, or dreams?* As another example, a question on self-blame and guilt in the original survey was replaced with a question on experiences of negative thoughts or moods associated with the pandemic. In addition to the five questions from the scale, an additional question assessed if the pandemic had caused any fear, helplessness, or horror among participants. Response choices were yes (1 point) or no (0 points). Scores ranged from 0 to 5 with scores greater than 4 signifying a positive PTSD screen. Cronbach’s alpha for our sample was 0.696.

#### 2.4.5 Emotional response to COVID-19

To further assess mental health associated with the pandemic, four items gathered information about feelings of anxiety, worry, upset, and coping. These items were adapted from an online survey developed by the South African Depression and Anxiety Group (SADAG).^1^ An example item is *Because of the COVID-19 pandemic, I feel anxious.* Participants responded to items using a three-point Likert scale where 0 = *more than usual*, 1 = *less than usual*, and 2 = *the same*. Scores ranged from 1 to 4 with lower scores indicating more frequent negative mental health symptoms during the pandemic compared to pre-pandemic experiences. Cronbach’s alpha for our sample was 0.699.

#### 2.4.6 Activities during lockdown

Participants’ activities during the lockdown – routine activities, exercise, and COVID-19 news exposure – were assessed using seven items adapted from the SADAG online survey of COVID-19 and mental health (SADAG). One item asked participants to indicate whether they engaged in the following activities during lockdown *never, daily, weekly,* or *monthly*: kept to a routine; exercised; changed clothes; spoke with someone outside the home via telephone or virtually; watched television or movies for entertainment; read/watched updates for COVID-19; and tried a new activity (e.g., online course, baking, and reading). Other items assessed respondents’ perception that they needed mental health assistance (*yes/no*); if so, why (*stress, anxiety, depression, substance use, other*) and to whom they reached out for mental health support (*a trusted friend/family member, free online support, paid counseling services, other*); how often they considered seeking assistance (*never, occasionally, frequently*); whether they sought assistance (*yes/no*); and, if so, if they sought assistance from a registered mental health professional (*yes/no*). Cronbach’s alpha for our sample was 0.510. Although relatively low, the use of these items is justified given the small number of items; the fact that the items assess different activities, which may not be correlated with one another; and the exploratory nature of this study.

### 2.5 Data analysis

Data were analyzed using the IBM Statistical Package for the Social Sciences version 26. Descriptive analyses were used to describe the sample and variables of interest (e.g., mental health, activities during lockdown). Authors conducted normality testing of the data.Results of the Kolmogorov–Smirnov and Shapiro–Wilk tests suggested that the data was not normally distributed, thus we conducted nonparametric testing to determine relationships between variables. The Mann Whitney U Test and Kruskal –Wallis Test were used in place of Independent Samples T-tests and One-way ANOVA. To address small subsamples (e.g., students identifying as transgender), the Kruskal-Wallis test was used. *Post hoc* tests (Dunn’s) were used to determine the direction of significant differences seen across groups. Correlation between mental health variables was assessed using Kendall’s tau-b. Given the low number of missing data values, all missing data were excluded from analysis.

## 3 Results

### 3.1 Participant demographics

Most participants (61.5%, *n* = 429) were aged 20–29 years. Most were women (75.8%, *n* = 526) and identified as White (79.5%, *n* = 554) followed by Black/African American (10% *n* = 70) and Asian (7.2%, *n* = 50). Most identified as non-Hispanic (82.1%, *n* = 572). Most participants were completing a Bachelor’s degree (64.3%, *n* = 448) across a wide variety of majors and reported a monthly income of $1,000 or less (61.8%, *n* = 431).

Most participants (83.4%, *n* = 581) did not report food insecurity as a result of the pandemic. Further, most participants (70.7%, *n* = 493) did not live in the residence halls and experienced no disruption in housing. However, among the 176 respondents who lived in the residence halls, 54% (*n* = 96) reported having to relocate during the COVID-19 pandemic.

Nearly 40% (*n* = 268) of participants had previously been diagnosed with a mental illness. Commonly cited diagnoses were General and Social Anxiety Disorder, Major Depressive Disorder, Attention Deficit Hyperactivity Disorder, Panic Disorder, Post-Traumatic Stress Disorder, Obsessive Compulsive Disorder, and Bipolar Disorder. The vast majority of participants (92.7%, *n* = 646) had not received COVID-19 testing at the time of data collection. See Table 1.

### 3.2 Mental health during the COVID-19 pandemic

#### 3.2.1 General health

Given the non-parametric nature of our data, we report median (MDN) and interquartile ranges (IQR) as comparison points. The median total score on the GHQ was 16.00, *IQR* = 10.00. A significant portion (46.6%, *n* = 297) of participants scored below the threshold for stress (<16); 132 (20.7%) scored between 16–20, indicating stress; and 208 (32.7%) scored >20, demonstrating severe psychological distress. Using the Kruskal-Wallis Test, significant differences were noted between GHQ scores and gender, *H*(2) = 16.41, *p* < 0.001. Pairwise comparisons using Dunn’s *post hoc* test indicated that cis gender men, *MDN* = 13.00, *IQR* = 10.00 had better outcomes than women *MDN* = 17.00, *IQR* = 10.00, *p* = 0.002 and transgender men, *MDN* = 25, *IQR* = 6.50, *p* = 0.02. To ensure the small number of transgender men in our sample did not distort findings, we re-ran the analyses without transgender men and found similar results. Specifically, results from the Mann Whitney U test showed that women had significantly worse general health (higher GHQ scores) compared to men, *z* = −3.245, *p* = 0.001. There were no significant differences by age groups, *p* = 0.33 or by race (*p* values ranged from 0.35–0.77) or ethnicity, *p* = 0.99. There were also no differences based on a previous mental health diagnosis, *p* = 0.56.

#### 3.2.2 Primary care post traumatic stress disorder screen (PC-PTSD)

The median score on the PC-PTSD was 2.00 and approximately one fourth of participants (25.7%, *n* = 167) had a score greater than 4, which is the threshold for a positive PTSD screen, as noted above. There was a significant difference in scores based on gender *H*(2) = 23.20, *p* < 0.001. Dunn’s *post hoc* results showed both women, *MDN* = 2.00, *IQR* = 3.00, *p* < 0.0001 and transgender men, *MDN* = 5, *IQR* = 2.00, *p* = 0.002 had higher PTSD scores than cis gender men, *MDN* = 1.00, *IQR* = 3. To ensure the small number of transgender men in our sample did not distort findings, we re-ran the analyses without transgender men and found similar results. Specifically, results from the Mann Whitney U test showed that women had significantly worse PTSD scores compared to men, *z* = −3.898, *p* < 0.001. There were no significant differences in scores based on age groups, *p* = 0.17 or by race (*p*-values ranged from 0.18–0.53) or ethnicity, *p* = 0.86. There was a significant difference based on previous mental health diagnosis. Participants with a previous mental health diagnosis reported worse PTSD scores, *MDN* = 3.00, *IQR* = 2.00 compared to participants without a history, *MDN* = 2.00, *IQR* = 3.00, *p* < 0.001.

#### 3.2.3 Emotional response to COVID-19

There was a significant difference in mental symptoms based on gender, *H*(2) = 16.126, *p* < 0.001. Dunn’s *post hoc* results showed cis gender men, *MDN* = 2.00, *IQR* = 1.19 reported better mental health experiences than women, *MDN* = 1.75, *IQR* = 1.00, *p* = 0.005 and transgender men, *MDN* = 1.00, *IQR* = 0.25, *p* = 0.008. To ensure the small number of transgender men in our sample did not distort findings, we re-ran the analyses without transgender men and found similar results. Specifically, results from the Mann Whitney U test showed that men had better emotional responses to COVID-19 compared to women, *z* = −3.413, *p* = 0.002. There were no significant differences in scores based on age, *p* = 0.13 or by race (*p*-values ranged from 0.44–0.90) or ethnicity, *p* = 0.49. However, there was a significant difference based on previous mental health diagnosis. Participants without a previous mental health diagnosis reported a more positive emotional response to COVID, *MDN* = 2.00, *IQR* = 1.00 = compared to participants with a history of mental health issues, *MDN* = 1.5, *IQR* = 1.00, *p* < 0.001.

Over one-third of respondents (37%, *n* = 258) reported needing mental health support because of the pandemic, including the lockdown. The most cited reasons for needing mental health support were anxiety (34%, *n* = 237), stress (32.9%, *n* = 229), and depression (27.7%, *n* = 193). The most frequently reported source of support (44%, *n* = 307) was a trusted friend/family member. Over half of participants (54.6%, *n* = 381) considered seeking professional mental health care on occasion (38.7%, *n* = 270) or frequently (15.9%, *n* = 111). However, only 22.7% of respondents sought assistance. Among these, 56.9% (*n* = 90) sought care from a professional mental health provider or hotline.

### 3.3 Relationship between mental health variables

Results showed a significant, moderate inverse correlation between general health and participants’ emotional response to COVID-19 (tb = −0.49, *p* < 0.001). Poor general health reports were correlated with a more negative emotional response to COVID-19 including feeling anxious or worried. There also was a significant correlation between general health and PTSD symptoms (tb = 0.55, *p < 0*.001). Specifically, worse GHQ scores. Mirrored worse PTSD scores. See Table 2.

**Table 2:** Correlations between variables.

Measure	1	2
1. General health		
2. COVID-emotional response	−0.604***	
3. PTSD	0.668***	−0.618***

***p < 0.001.

### 3.4 Activities during COVID-19 lockdown

Most participants reported maintaining a daily or weekly routine (67.0%, *n* = 467), changing their clothes daily (74.2%, *n* = 517), speaking with others outside the home by telephone or other virtual methods (e.g., Skype or Zoom) daily or weekly (74.5%, *n* = 519), watching television or movies for entertainment daily (61.4%, *n* = 428), and reading or watching updates on COVID-19 daily or weekly (73.6%, *n* = 513). Approximately half of participants (50.9%, *n* = 355) exercised daily or weekly. Less than half of participants (40.8%, *n* = 284) reported trying a new activity daily or weekly. See Table 3.

**Table 3:** Lockdown activities in percentages.

Measure	Daily	Weekly	Monthly	Never
Kept a routine	31.6% (220)	35.4% (247)	4.7% (32)	12.9% (90)
Exercised	19.8% (138)	31.1% (217)	17.6 (123)	16.1 (112)
Changed clothes	74.2% (517)	9.2% (64)	0.4% (3)	0.9% (6)
Spoke with someone outside the home through telephone, Skype, etc.	46.1% (321)	28.4% (198)	8.0% (38)	2.2% (14)
Watched TV, movies for entertainment	61.4% (428)	18.2% (127)	3.3% (22)	1.7% (11)
Reading, watching updates on COVID	43.0% (300)	30.6% (213)	6.2% (39)	4.9% (33)
Tried a new activity	14.8% (103)	26.0% (181)	22.5% (157)	21.4% (149)

### 3.5 Impact of activities during COVID-19 lockdown on mental health

Authors also examined the relationship between lockdown activities during the COVID-19 pandemic and mental health outcomes. There were differences in mental health outcomes based on the extent to which participants had a routine, exercised, and were exposed to COVID-related information.

#### 3.5.1 Routine activities and mental health

According to findings of the Kruskal-Wallis Test, there was a significant difference in GHQ-12 scores based on routine frequency at the *p* < 0.05 level, *H*(3) = 46.76, *p* < 0.001. Dunn’s *post hoc* test showed that participants with a daily routine, *MDN* = 15.00, *IQR* = 9.00 had better outcomes compared to participants with a weekly routine, *MDN* = 17.00, *IQR* = 9.00, *p* = 0.009, monthly routine, *MDN* = 18.00, *IQR* = 9.00 *p* = 0.026, or no routine, *MDN* = 22.50, *IQR* = 12.25, *p* < 0.0001. Those with a weekly routine also fared better than those without a routine, *p* < 0.0001.

There was a significant difference in PTSD scores based on routine frequency at the *p* < 0.05 level, *H*(3) = 19.89, *p* < 0.001. Participants with a daily routine, *MDN* = 2.00, *IQR* = 2.00 had better outcomes than participants without a routine, *MDN* = 3.00, *IQR* = 2.00 *p* < 0.0001.

There was a significant difference in emotional response scores based on routine frequency at the *p* < 0.05 level, *H*(3) = 19.67, *p* < 0.001. Participants with a daily routine, *MDN* = 2.00, *IQR* = 1.00 had better emotional responses than participants with a weekly *MDN* = 2.00, *IQR* = 1.25, *p* = 0.048, monthly *MDN* = 1.50, *IQR* = 1.00, *p* = 0.006, or no routine, *MDN* = 1.50, *IQR* = 1.25 *p* = 0.004.

#### 3.5.2 Exercise and mental health

There were significant differences in GHQ-12 scores based on having an exercise habit at the *p* < 0.05 level, *H*(3) = 18.958, *p* < 0.003. Participants who exercised daily, *MDN* = 14.00, *IQR* = 11.00 had better outcomes than participants who exercised monthly, *MDN* = 17.00*IQR* = 9.00, *p* = 0.005, or not at all, *MDN* = 18.5, *IQR* = 11.25, *p = 0*.0001.

There were significant differences in PTSD scores based on exercise habits at the *p* < 0.05 level, *H*(3) = 10.380, *p* = 0.018. Participants who exercised monthly, *MDN* = 3.00, *IQR* = 3.00 had worse PTSD scores compared to participants who exercised daily, *MDN* = 2.00, *IQR = 2.00*, *p* = 0.04.

There were no significant differences in emotional response to COVID-19 based on exercise frequency, *p* = 0.10.

#### 3.5.3 COVID-19 news exposure and mental health

There was a significant difference in GHQ-12 scores based on frequency of watching/reading COVID updates at the *p* = 0.05 level, *H*(3) = 11.5423, *p* = 0.010. Participants who viewed daily content on COVID-19 had poorer general health outcomes, *MDN* = 17.00, *IQR* = 10.00 compared to participants who never viewed any such content, *MDN* = 14, *IQR* = 8.50 *p* = 0.03. There was a significant difference in PTSD scores based on frequency of watching/reading COVID updates at the *p* < 0.05 level, *H*(3) = 22.242, *p* < 0.009. Participants who watched media content on COVID daily had worse outcomes, *MDN* = 3.00, *IQR* = 3.00 compared to participants who watched weekly, *MDN* = 2.00, *IQR* = 2.00, *p* = 0.002, monthly, *MDN* = 2.00, *IQR* = 3.00, *p* = 0.010, or never, *MDN* = 2.00, *IQR* = 3.00, *p* = 0.04.

There was a significant difference in emotional response scores based on frequency of watching/reading COVID updates at the *p* < 0.05 level, *H*(3) = 31.626; *p* < 0.001. Participants who watched/read daily updates, *MDN* = 1.50, *IQR* = 1.00 had poorer emotional responses compared to participants who watched weekly, *MDN* = 2.00, *IQR* = 1.00, *p* < 0.0001, or never, *MDN* = 2.5, *IQR* = 1.00, *p* < 0.0001. Other behaviors such as changing clothes, speaking to someone outside the home, and watching tv or movies did not show any relationship with changes in PTSD, GHQ or emotional response.

## 4 Discussion

Current findings provide data on COVID-19’s mental health impact among college students in the southeastern US, a setting considered particularly vulnerable to COVID-19 (27) and where access to mental health treatment is limited compared to other US regions (28). Several factors were associated with students’ mental health early in the pandemic. Being a woman or a transgender man, pre-existing mental health conditions, low engagement in routine activities, and COVID-19 news exposure were associated with college student mental health. These findings, and others, are described in more detail below.

### 4.1 Gender and mental health during COVID-19

In this study, more women reported adverse mental health outcomes associated with the pandemic followed by transgender men. The finding that women were more negatively affected by COVID-19 is consistent with findings from a systematic review and meta-analysis, which revealed higher levels of COVID-related psychological distress among women compared to men in the general population (5). However, while findings from some individual studies examining COVID-19’s impact on college students, specifically, provide evidence of more negative mental health outcomes for women students [e.g., Xu et al. (38)], a systematic review and meta-analysis of studies focused on COVID’s mental health impact on college students revealed no gender differences in anxiety or depression (2). In another systematic review focused on the mental health impact of COVID-19 on college students, gender differences were not examined (4).

Despite mixed findings regarding gender differences in mental health impact related to COVID-19, the finding that women were more negatively affected than men in the current study is not surprising given that gender differences in mental health are well established (39). Less data exists on the differential mental health impact of COVID-19 on students based on sexual orientation and gender identity, thus current findings contribute something important to the existing literature. However, current results support findings from Gonzales et al.’s study (40), which described the mental health needs of students who identify as LGBTQ+. Indeed, the mental health needs of people who identify as transgender has been documented (41). Combined, findings suggest gender is an important mental health determinant, thus prompting a need for interventions in the college setting that target women and gender minorities.

### 4.2 Race/ethnicity and mental health during COVID-19

Inconsistent with other studies, current findings did not provide evidence of a differential impact of COVID-19 on mental health based on race or ethnicity. Xu et al. (38) found COVID-19’s mental health impact on college students was greater for Hispanic students compared to other ethnic groups. Molock and Percham (42) described symptoms of stress, anxiety, and depression associated with COVID-19 among students of color as well as other negative effects (e.g., disruptions in living situation, finances, academic performance, educational plans, and career goals). Students in Molock and Percham’s study (42) also described difficulties managing feelings surrounding racial injustice, noting that George Floyd’s killing early in the pandemic heightened awareness of racism in the US, thus compounding mental health challenges during COVID-19. Molock and Percham (42) did not compare the mental health of students of color to White students’ mental health so conclusions cannot be drawn about the differential impact of COVID-19 on mental health between these groups. The absence of mental health differences based on race or ethnicity in this study may be attributed, at least in part, to the student body at the university where data was collected being ranked in the top 15% for racial/ethnic diversity among the 3,790 universities included in the rankings (43).

### 4.3 Pre-existing conditions and mental health during COVID-19

Nearly 40 percent of students in this study reported a pre-existing mental health condition. Participants reporting previous diagnoses also reported worse mental health outcomes, which is not surprising. Asmundson et al. (44) showed pre-existing mental health problems affect coping and general response to stress. This finding was more the case for individuals who self-reported anxiety related disorders than those who self-reported mood disorders (44). While the current study does not show such distinctions, findings do demonstrate COVID-19 had a worse impact on students with pre-existing mental illness(es) compared to students with no history of mental illness, thus highlighting the need for specialized services in the college setting for students living with diagnosed mental health conditions.

Despite the significant mental health challenges posed by the pandemic on students and the high number of students who had pre-existing conditions, a little over one third of participants reported needing mental health support. From that third, only about half considered seeking help and just 27% sought assistance. This finding demonstrates a pattern of underreporting and poor health seeking behavior among college students despite high levels of need. These results are consistent with the literature on decreased health seeking behavior during the COVID-19 pandemic (45, 46). Identifying ways to increase mental health service uptake among college students is therefore important.

### 4.4 Routine activities and mental health during COVID-19

Students reported creating new, or maintaining old, routines such as exercising or leisure activities like reading books and watching television, which may have been a means of coping during the pandemic. Results showed students who engaged in activities such as exercising more frequently had better mental health outcomes than students who engaged in such activities less frequently or not at all. These findings are consistent with Rogowska *et al*’s report of a relationship (47), albeit weak, between physical activity and symptoms of anxiety and depression among a large sample of Ukrainian university students. However, other research in this area is mixed. Findings from a longitudinal cohort study in the United Kingdom (48) demonstrated a relationship, albeit weak, between perceived stress and sedentary behavior among college students. However, Savage et al. (48) did not find a relationship between mental health and moderate to vigorous physical activity although both decreased during the pandemic. Similarly, Talapko et al. (49) did not find a relationship between decreased physical activity and mental health symptoms of Croatian university students, nor did Wilson et al. (50) find physical activity served as a protective factor against COVID-19 related mental health issues among college students at a northeastern university in the US. Given mixed findings in this area, more research is needed to explore the impact of exercise on mental health among college students during COVID-19.

Beyond exercise, current findings suggest leisure activities may be beneficial for mental health. Some research has demonstrated that the relationship between leisure activities and mental health depends on the type of leisure activity. For example, reading may have mental health benefits whereas online activities may not. Research indicates that resilience may mediate the relationship between leisure activities and mental health, which is consistent with the broaden-and-build theory that suggests positive emotions associated with leisure activities may increase the mental resources needed to cope with stressful events (51).

### 4.5 Exposure to COVID-19 news and mental health

Exposure to COVID-19 information presented a coping challenge for students. Students who were less active on social media during the pandemic had a better chance of avoiding misinformation that might cause anxiety. Studies have shown COVID-19 information disseminated through social media increased levels of panic and general anxiety especially among younger people (52, 53). With young people more likely to get information from social media than from other verifiable or official government sources, discerning between true and false information becomes difficult. Media coverage has been extensive during the COVID-19 pandemic, which has been vital to informing the public about prevention measures. However, such coverage can easily raise fear and panic. Although little research exists on the mental health impact of news exposure on college students, specifically, a growing body of literature supports the negative impact of news exposure on the mental health among the general population (54–56).

## 5 Limitations

This study had several limitations. Although the exploratory nature of this study and the use of a cross-sectional design were justified given that little data on COVID-19 existed at the time of data collection and randomization was not possible, the cross-sectional design limits the ability to make causal inferences and to account for confounding variables. Also, although this study intentionally focused on the southeastern region of the US, data was limited to one large public university and may not generalize to other universities in this region. Further, data are based on self-report, thus introducing response bias as a potential issue although the use of an anonymous online survey should mitigate this concern. The sample was over-represented by women, which may affect the applicability of findings to people with other gender identities. Nonetheless, given that women outnumber men on college campuses (57), understanding the experiences of women college students is prudent. Related to the issue of sampling, the small number of transgender men in our sample may be viewed as a weakness. Although we re-ran our analyses without transgender men to confirm the small number did not distort our findings (see results section above), the small number of transgender men in our sample may limit the generalizability of findings to other college students who identify as transgender men. Despite these concerns, the inclusion of transgender men in our analyses is justified by the need to better understand the unique experiences of people who identify as transgender. Additionally, excluding people who identify as transgender from research perpetuates their invisibility and promotes marginalization, which are contrary to the promotion of equitable and inclusive research practices. Finally, the measures used in this study pose limitations. There is considerable item overlap between the GHQ-12 and the SADAG, which likely contributes to the strong correlation between scores on those measures. Also, the adaptation of scales assessing the effect of COVID-19 may limit findings. Specifically, the low internal consistency of some measures raises questions about construct validity and the possibility of measuring more than one latent variable. However, lower alphas in our sample for some measures may be a function of a small number of items or the lack of unidimensionality (e.g., the items assessing activities during lockdown assessed several different dimensions). Therefore, a lower alpha should not render a measure unreliable in our sample.

## 6 Conclusion and future directions

In conclusion, this exploratory study provides valuable insights into the mental health impact of COVID-19 among college students in the southeastern US, a region considered vulnerable to COVID-19 and with limited access to mental health treatment compared to other regions. Several factors were associated with students’ mental health during the early stages of the pandemic, including gender, pre-existing mental health conditions, engagement in routine activities, and exposure to COVID-19 news. Women and transgender men were more adversely affected by the pandemic, highlighting the importance of gender-specific interventions in the college setting. While the study did not find significant differences in mental health impact based on race or ethnicity, the need for specialized services for students with pre-existing mental health conditions was evident. Engaging in routine activities, especially exercise and leisure activities, appeared to have a positive impact on mental health outcomes. However, exposure to COVID-19 news posed a coping challenge, with those less active on social media experiencing less anxiety. This study underscores the importance of addressing mental health needs of students during the pandemic and highlights the need for further research to inform effective interventions. It also emphasizes the significance of accurate information dissemination and health-seeking behavior among college students to promote their well-being.

Although other research has documented similar findings with regard to factors affecting college students’ mental health during COVID-19, this study’s focus on the southeastern U.S. contributes to our understanding of regional similarities and differences. Further, findings provide some emerging insights into the stress and coping response of transgender students during the pandemic.

As research in this area continues to emerge, colleges and universities are encouraged to adapt mental health programming to better meet students’ needs including targeting at-risk students for prevention and intervention programs. Based on findings from a study of factors associated with depression, anxiety, and PTSD among young adults in the US, Liu et al. (58) emphasized the importance of family support and suggested mental health programming for young people should focus on decreasing loneliness and improving distress tolerance. Although ensuring family support may be difficult for higher education institutions, developing programs to promote social support and build resilience are within their purview. Building upon Roksa and Kinsley’s (59) suggestions for fostering family support for low income students, two specific recommendations are offered to administrators in higher education for promoting family support, especially for students at risk for negative mental health outcomes: (1) engage in dialogue with parents from diverse backgrounds (e.g., based on socioeconomic status, country or region of origin) and with diverse identities (e.g., based on gender identity, sexual orientation, race, ethnicity, age) about the social–emotional and academic needs of their college-aged children; and (2) educate faculty, advisors, and staff about the importance of family support for student success.

In a longitudinal study with timepoints before, during, and after the COVID-19 lockdown, Li et al. (60) found novelty seeking, defined as behaviors related to the pursuit of new experiences, was associated with decreased symptoms of anxiety, stress, and depression among Chinese university students. As Li et al. (60) noted, novelty underlies creativity and novelty seeking may mitigate the negative mental health impact of crises such as the COVID-19 pandemic. Findings such as these also may be useful when developing strategies to support college students’ mental health needs. For example, An et al. (61) recommend interventions that teach and promote the use of active coping strategies in order to reduce student stress.

Future research should further explore COVID-19’s impact on college students, particularly long-term mental health effects as well as academic progress and career trajectories. Also needed is research examining the effectiveness of interventions designed to treat college students experiencing mental health problems associated with COVID-19. Finally, research is needed on the long-term impact of COVID-19 on educational practices in higher education and how enduring changes prompted by COVID-19 (e.g., virtual learning) will affect college students’ mental health long past this pandemic’s end. Research designs that allow for the control of confounding variables, such as randomized controlled trials, will be important when possible.

## Ethics statement

This study involved humans and was approved by the University of South Florida Institutional Review Board (TC). The study was conducted in accordance with local legislation and institutional requirements. The ethics committee/institutional review board waived the requirement of written informed consent for participation from the participants because the study used an online anonymous survey. No identifying information was collected. Participants were notified that by entering the online survey they were consenting to participate in the study.

## Author contributions

TC was responsible for study design, investigation, interpreting data, and wrote first and subsequent drafts. KG analysed and interpreted data, assisted in original draft writing, and draft review and editing. CZ contributed to draft writing and review and editing. SH reviewed first and subsequent drafts. JD was responsible for study design and conceptualization, investigation, and review of drafts and editing. All authors contributed to the article and approved the submitted version.

## Footnotes

^1^This study was part of a larger cross-cultural study designed to examine differences in student mental health in response to the COVID-19 pandemic between the US and South Africa. Because the original project was designed by South African authors, some of the measures used in this study originated in South Africa.

## References

[1] Ćosić K Popović S Šarlija M Kesedžić I. Impact of human disasters and COVID-19 pandemic on mental health: potential of digital psychiatry. Psychiatr Danub. (2020) 32:25–31. doi: 10.24869/psyd.2020.25, PMID: 32303026

[2] Li Y Wang A Wu Y Han N Huang H. Impact of the COVID-19 pandemic on the mental health of college students: a systematic review and meta-analysis. Front Psychol. (2021) 12:669119. doi: 10.3389/fpsyg.2021.669119, PMID: 34335381 PMC8316976

[3] Vindegaard N Benros ME. COVID-19 pandemic and mental health consequences: systematic review of the current evidence. Brain Behav Immun. (2020) 89:531–42. doi: 10.1016/j.bbi.2020.05.048, PMID: 32485289 PMC7260522

[4] Wang C Wen W Zhang H Ni J Jiang J Cheng Y. Anxiety, depression, and stress prevalence among college students during the COVID-19 pandemic: a systematic review and meta-analysis. J Am Coll Health. (2021) 1:1–8. doi: 10.1080/07448481.2021.1960849, PMID: 34469261

[5] Wang Y Kala MP Jafar TH. Factors associated with psychological distress during the coronavirus disease 2019 (COVID-19) pandemic on the predominantly general population: a systematic review and meta-analysis. PLoS One. (2020) 15:e0244630. doi: 10.1371/journal.pone.0244630 33370404 PMC7769562

[6] Goldmann E Galea S. Mental health consequences of disasters. Annu Rev of Public Health. (2014) 35:169–83. doi: 10.1146/annurev-publhealth-032013-182435 24159920

[7] Beaglehole B Mulder RT Frampton CM Boden JM Newton-Howes G Bell CJ. Psychological distress and psychiatric disorder after natural disasters: systematic review and metaanalysis. Br J Psychiatry. (2018) 213:716–22. doi: 10.1192/bjp.2018.210, PMID: 30301477

[8] Zhou S-J Zhang L-G Wang L-L Guo ZC Wang JQ Chen JC. Prevalence and sociodemographic correlates of psychological health problems in Chinese adolescents during the outbreak of COVID-19. Eur Child Adolesc Psychiatry. (2020) 29:749–58. doi: 10.1007/s00787-020-01541-4, PMID: 32363492 PMC7196181

[9] Rehm J Shield KD. Global burden of disease and the impact of mental and addictive disorders. Curr Psychiatry Rep. (2019) 21:10. doi: 10.1007/s11920-019-0997-0 30729322

[10] Shigemura J Ursano RJ Morganstein JC Kurosawa M Benedek DM. Mental health in the post-COVID-19 era: challenges and the way forward. Psychiatry Clin Neurosci. (2020) 74:281–2. doi: 10.1111/pcn.12988, PMID: 32034840 PMC7168047

[11] Hossain MM Tasnim S Sultana A Faizah F Mazumder H Zou L. Epidemiology of mental health problems in COVID19: a review. F1000Res. (2020) 9:1–16. doi: 10.12688/f1000research.24457.1, PMID: 33093946 PMC7549174

[12] Cénat JM Dalexis RD Kokou-Kpolou CK Guerrier M Rousseau C. Social inequalities and collateral damages of the COVID-19 pandemic: when basic needs challenge mental health care. Int J Public Health. (2020) 65:717–8. doi: 10.1007/s00038-020-01426-y, PMID: 32651593 PMC7348102

[13] de Miquel C Domènech-Abella J Felez-Nobrega M Cristóbal-Narváez P Mortier P Vilagut G. The mental health of employees with job loss and income loss during the COVID19 pandemic: the mediating role of perceived financial stress. Int J Environ Res Public Health. (2022) 19:3158. doi: 10.3390/ijerph19063158, PMID: 35328846 PMC8950467

[14] Yan Y Wang X Zhang R Zhang Y. The relationship between leisure activities and the mental health of college students during the COVID-19 pandemic. World Leisure J. (2023) 65:119–42. doi: 10.1080/16078055.2022.2158924

[15] Zhang X. Household chaos and caregivers’ and young children’s mental health during the COVID-19 pandemic: a mediation model. J Child Fam Stud. (2022) 31:1547–57. doi: 10.1007/s10826-022-02283-4, PMID: 35287306 PMC8907899

[16] Varma P Junge M Meaklim H Jackson ML. Younger people are more vulnerable to stress, anxiety and depression during COVID-19 pandemic: a global cross-sectional survey. Prog Neuro-Psychopharmacol Biol Psychiatry. (2021) 109:110236. doi: 10.1016/j.pnpbp.2020.110236, PMID: 33373680 PMC7834119

[17] Islam MA Barna SD Raihan H Khan MNA Hossain MT. Depression and anxiety among university students during the COVID-19 pandemic in Bangladesh: a web-based cross-sectional survey. PLoS One. (2020) 15:e0238162. doi: 10.1371/journal.pone.0238162, PMID: 32845928 PMC7449469

[18] Pedrelli P Nyer M Yeung A Zulauf C Wilens T. College students: mental health problems and treatment considerations. Acad Psychiatry. (2015) 39:503–11. doi: 10.1007/s40596-014-0205-9, PMID: 25142250 PMC4527955

[19] Wang X Hegde S Son C Keller B Smith A Sasangohar F. Investigating mental health of US college students during the COVID-19 pandemic: cross-sectional survey study. J Med Internet Res. (2020) 22:e22817. doi: 10.2196/22817 32897868 PMC7505693

[20] Son C Hegde S Smith A Wang X Sasangohar F. Effects of COVID-19 on college students’ mental health in the United States: interview survey study. J Med Internet Res. (2020) 22:e21279. doi: 10.2196/21279, PMID: 32805704 PMC7473764

[21] Frazier P Liu Y Asplund A Meredith L Nguyen-Feng VN. US college student mental health and covid-19: comparing pre-pandemic and pandemic timepoints. J Am Coll Heal. (2021):1–11. doi: 10.1080/07448481.2021.1987247, PMID: 34762560

[22] Islam MS Sujan MSH Tasnim R Sikder MT Potenza MN van Os J. Psychological responses during the COVID-19 outbreak among university students in Bangladesh. PLoS One. (2020) 15:e0245083. doi: 10.1371/journal.pone.0245083, PMID: 33382862 PMC7775049

[23] Commodari E Rosa VLL Carnemolla G Parisi J. The psychological impact of the lockdown on Italian university students during the first wave of COVID-19 pandemic: Psychological experiences, health risk Perceptions, distance learning, and future perspectives. MJCP. (2021) 9. doi: 10.13129/2282-1619/mjcp-3009

[24] La Rosa VL Commodari E. University experience during the first two waves of COVID-19: Students' experiences and psychological wellbeing. Eur J Investig Health Psychol Educ. (2023) 13:1477–90. doi: 10.3390/ejihpe13080108, PMID: 37623305 PMC10453088

[25] Wathelet M Duhem S Vaiva G. Factors associated with mental health disorders among university students in France confined during the COVID-19 pandemic. JAMA Netw Open. (2020) 3:e2025591. doi: 10.1001/jamanetworkopen.2020.25591 33095252 PMC7584927

[26] Soria KM Horgos B. Factors associated with college students' mental health during the COVID-19 pandemic. J Coll Stud Dev. (2021) 62:236–42. doi: 10.1353/csd.2021.0024

[27] Johnston CD Chen R. The COVID-19 pandemic and its impact on the southern United States. J Comp Fam Stud. (2020) 51:314–23. doi: 10.3138/jcfs.51.3-4.007

[28] America MH. 2019 state of mental health in America report. (2018). Available at: https://www.mhanational.org/research-reports/2019-state-mental-health-america-report (Accessed December 26, 2022)

[29] Prevention CfDCa. COVID-19 mortality by state. Ctr Dis Control Prev. Available at: https://www.cdc.gov/nchs/pressroom/sosmap/covid19_mortality_final/COVID19.htm

[30] Bureau USC. Census regions and divisions of the United States. Available at: https://www2.census.gov/geo/pdfs/maps-data/maps/reference/us_regdiv.pdf

[31] Services USCfMM. COVID-19 vaccination rates – state and national averages. Data.CMS.gov. Available at: https://data.cms.gov/provider-data/dataset/avax-cv19

[32] Organization WH. WHO Coronavirus (COVID-19) dashboard. World Health Organization. Available at: https://covid19.who.int/table/

[33] Factual C. [Institution blinded for review] diversity: Racial demographics & other stats, (2022). Available at: https://www.collegefactual.com/colleges/university-of-south-florida-st-petersburg-campus/student-life/diversity/

[34] Hanson M. College enrollment statistics [2022]: Total + by demographic. Education Data Initiative, (2022). Available at: https://educationdata.org/college-enrollment-statistics

[35] Bureau USC. Race/ethnicity and the 2020 census. (2022). Available at: https://www.census2020now.org/faces-blog/same-sex-households-2020-census-r3976

[36] Goldberg DP Williams P. A user's guide to the general health questionnaire. London: GL Assessment (2006).

[37] Prins A Bovin MJ Smolenski DJ Marx BP Kimerling R Jenkins-Guarnieri MA. The primary care PTSD screen for DSM-5 (PC-PTSD-5): development and evaluation within a veteran primary care sample. J Gen Intern Med. (2016) 31:1206–11. doi: 10.1007/s11606-016-3703-5, PMID: 27170304 PMC5023594

[38] Xu L Lu W Smith AW Wu Q Chan V Hou A. Gender, ethnicity differences in mental health status and COVID-19 vaccine intention among U.S. college students during COVID-19 pandemic. J Am Coll Heal. (2022):1–10. doi: 10.1080/07448481.2022.2070024 35549991

[39] Afifi M. Gender differences in mental health. Singap Med J. (2007) 48:385–91. PMID: 17453094

[40] Gonzales G Loret de Mola E Gavulic KA McKay T Purcell C. Mental health needs among lesbian, gay, bisexual, and transgender college students during the COVID-19 pandemic. J Adolesc Health. (2020) 67:645–8. doi: 10.1016/j.jadohealth.2020.08.006, PMID: 32933837 PMC10251764

[41] McCann E Sharek D. Mental health needs of people who identify as transgender: a review of the literature. Arch Psychiatr Nurs. (2016) 30:280–5. doi: 10.1016/j.apnu.2015.07.003, PMID: 26992883

[42] Molock SD Parchem B. The impact of COVID-19 on college students from communities of color. J Am Coll Heal. (2021) 70:2399–405. doi: 10.1080/07448481.2020.1865380, PMID: 33502970

[43] Factual C. University blinded for review – Main campus diversity: Racial demographics & other stats. Available at: https://www.collegefactual.com/colleges/university-of-south-florida-main-campus/student-life/diversity/#ethnic_diversity

[44] Asmundson GJG Paluszek MM Landry CA Rachor GS McKay D Taylor S. Do pre-existing anxiety-related and mood disorders differentially impact COVID-19 stress responses and coping? J Anxiety Disord. (2020) 74:102271–6. doi: 10.1016/j.janxdis.2020.102271, PMID: 32673930 PMC7342169

[45] Nab M van Vehmendahl R Somers I Schoon Y Hesselink G. Delayed emergency healthcare seeking behaviour by Dutch emergency department visitors during the first COVID-19 wave: a mixed methods retrospective observational study. BMC Emerg Med. (2021) 21:56. doi: 10.1186/s12873-021-00449-9 33932988 PMC8087882

[46] Aklilu TM Abebe W Worku A. The impact of COVID-19 on care seeking behavior of patients at tertiary care follow-up clinics: a cross-sectional telephone survey. Addis Ababa, Ethiopia. medRxiv. (2020):1–29. doi: 10.1101/2020.11.25.20236224

[47] Rogowska AM Pavlova I Kuśnierz C Ochnik D Bodnar I Petrytsa P. Does physical activity matter for the mental health of university students during the COVID-19 pandemic? J Clin Med. (2020) 9:3494. doi: 10.3390/jcm9113494 33138047 PMC7693909

[48] Savage MJ James R Magistro D Donaldson J Healy LC Nevill M. Mental health and movement behaviour during the COVID-19 pandemic in UK university students: prospective cohort study. Ment Health Phys Act. (2020) 19:100357. doi: 10.1016/j.mhpa.2020.100357

[49] Talapko J Perić I Vulić P Pustijanac E Jukić M Bekić S. Mental health and physical activity in health-related university students during the COVID-19 pandemic. Healthcare. (2021) 9. doi: 10.3390/healthcare9070801, PMID: 34202384 PMC8304952

[50] Wilson OWA Holland KE Elliott LD Duffey M Bopp M. The impact of the COVID-19 pandemic on US college students’ physical activity and mental health. J Phys Act Health. (2021) 18:272–8. doi: 10.1123/jpah.2020-0325, PMID: 33601332

[51] Takiguchi Y Matsui M Kikutani M Ebina K. The relationship between leisure activities and mental health: the impact of resilience and COVID-19. Appl Psychol Health Well Being. (2023) 15:133–51. doi: 10.1111/aphw.12394, PMID: 35971651 PMC9538683

[52] Ahmad AR Murad HR. The impact of social media on panic during the COVID-19 pandemic in Iraqi Kurdistan: online questionnaire study. J Med Internet Res. (2020) 22:e19556. doi: 10.2196/19556, PMID: 32369026 PMC7238863

[53] Radwan E Radwan A Radwan W. The role of social media in spreading panic among primary and secondary school students during the COVID-19 pandemic: An online questionnaire study from the Gaza strip, Palestine. Heliyon. (2020) 6:e05807. doi: 10.1016/j.heliyon.2020.e05807, PMID: 33376831 PMC7758520

[54] Kellerman JK Hamilton JL Selby EA Kleiman EM. The mental health impact of daily news exposure during the COVID-19 pandemic: ecological momentary assessment study. JMIR Ment Health. (2022) 9:e36966. doi: 10.2196/36966 35377320 PMC9135112

[55] Stainback K Hearne BN Trieu MM. COVID-19 and the 24/7 news cycle: does COVID-19 news exposure affect mental health? Socius. (2020) 6:237802312096933. doi: 10.1177/2378023120969339

[56] Shabahang R Aruguete MS McCutcheon LE. Online health information utilization and online news exposure as predictor of COVID-19 anxiety. N Am J Psychol. (2020) 22:469–82.

[57] (NCES) NCfES. Home page, a part of the U.S. department of Education, (2022). https://nces.ed.gov/fastfacts/display.asp?id=98

[58] Liu CH Zhang E Wong GTF Hyun S Hahm HC. Factors associated with depression, anxiety, and PTSD symptomatology during the COVID-19 pandemic: clinical implications for U.S. young adult mental health. Psychiatry Res. (2020) 290:113172. doi: 10.1016/j.psychres.2020.113172, PMID: 32512357 PMC7263263

[59] Roksa J Kinsley P. The role of family support in facilitating academic success of low-income students. Res High Educ. (2019) 60:415–36. doi: 10.1007/s11162-018-9517-z

[60] Li WW Yu H Miller DJ Yang F Rouen C. Novelty seeking and mental health in Chinese university students before, during, and after the COVID-19 pandemic lockdown: a longitudinal study. Front Psychol. (2020) 11. doi: 10.3389/fpsyg.2020.600739, PMID: 33343473 PMC7744699

[61] An H Chung S Park J Kim S-Y Kim KM Kim K-S. Novelty-seeking and avoidant coping strategies are associated with academic stress in Korean medical students. Psychiatry Res. (2012) 200:464–8. doi: 10.1016/j.psychres.2012.07.048, PMID: 22901439

